# Practicalities and Research Considerations for Conducting Childhood Obesity Prevention Interventions with Families

**DOI:** 10.3390/children3040024

**Published:** 2016-11-08

**Authors:** Philip J. Morgan, Rachel A. Jones, Clare E. Collins, Kylie D. Hesketh, Myles D. Young, Tracy L. Burrows, Anthea M. Magarey, Helen L. Brown, Trina Hinkley, Rebecca A. Perry, Leah Brennan, Alison C. Spence, Karen J. Campbell

**Affiliations:** 1 Priority Research Centre in Physical Activity and Nutrition, University of Newcastle, Callaghan Campus, Newcastle 2308, Australia; clare.collins@newcastle.edu.au (C.E.C.); myles.young@newcastle.edu.au (M.D.Y.); tracy.burrows@newcastle.edu.au (T.L.B.); 2 Early Start Research Institute, Faculty of Social Sciences, University of Wollongong, Wollongong 2522, Australia; rachelj@uow.edu.au; 3 Centre for Physical Activity and Nutrition Research, Deakin University, Geelong 3126, Australia; kylie.hesketh@deakin.edu.au (K.D.H.); h.brown@deakin.edu.au (H.L.B.); trina.hinkley@deakin.edu.au (T.H.); a.spence@deakin.edu.au (A.C.S.); karen.campbell@deakin.edu.au (K.J.C.); 4 Discipline of Nutrition and Dietetics, Faculty of Medicine, Nursing and Health Sciences, Flinders University, Adelaide 5042, Australia; anthea.magarey@flinders.edu.au (A.M.M.); r.perry@flinders.edu.au (R.A.P.); 5 School of Psychology, Australian Catholic University, Sydney 3065, Australia; drleahbrennan@gmail.com

**Keywords:** family-based, obesity, parents, prevention, interventions, child obesity prevention

## Abstract

Internationally, childhood obesity is a major public health concern. Given the established difficulties in treating obesity, designing and evaluating effective obesity prevention interventions are research priorities. As parents play a crucial role in establishing positive health behaviours in children, they are a key target for child obesity prevention programs. However, recruiting and engaging parents in such interventions can be a considerable challenge for researchers and practitioners. Members of the ‘Parenting, Child Behaviour and Well-being’ stream of the Australasian Child and Adolescent Obesity Research Network (ACAORN) have considerable and varied expertise in conducting such interventions and can provide insights into addressing these challenges. This paper aims to highlight considerations regarding the design, implementation, and evaluation of obesity prevention interventions with families and provide practical insights and recommendations for researchers and practitioners conducting family-based research in this area. Case studies of three family-based interventions conducted by ACAORN members are highlighted to provide examples and contextualise the recommendations proposed.

## 1. Introduction

Internationally, childhood obesity is a serious public health concern [1]. Currently, 24% of boys and 23% of girls in developed countries are overweight or obese [1], placing them at increased risk of both short- and long-term physical and psychological health consequences [2]. Obesity rates are also rising in developing countries [1], particularly amongst low socio-economic groups [3]. Given the recalcitrant nature of obesity once established [4], and research demonstrating that obesity tracks throughout life [5,6], evidence-based prevention interventions are needed.

The defining goal of obesity prevention interventions is to reduce weight gain or obesity risk in participants over time. However, it is important to note that obesity prevention interventions can also assist overweight participants to lose weight (or stabilise their weight and prevent progression to ‘obesity’). These interventions differ from obesity ‘treatment’ interventions, which focus exclusively on overweight or obese participants. Recent reviews have identified preliminary evidence for the effectiveness of existing obesity prevention interventions in children and youth [7,8]. However, the results of these interventions have been modest and most studies have been conducted in education settings such as preschools, primary, and high schools [7,8]. While schools are key settings for obesity prevention due to the captive audience they provide [9], school-based programs often fail to actively engage parents, who have a critical influence over their children’s health behaviours [10]. As such, interventions targeting parents to support positive physical activity and healthy eating behaviours in the home setting are vital [7].

Parents influence their children’s health behaviours through a host of mechanisms, including their general parenting style, parenting practices (e.g., role modelling, rule setting, behavioural consequences, and establishing behavioural expectations) and their control of home environment [11]. Thus, interventions which: (i) educate parents about the link between parenting practices and healthy lifestyle behaviours in children and (ii) help parents develop optimal parenting skills and knowledge, are likely to be of great benefit for parents and children alike. Despite this, it remains unclear how best to design, implement, and evaluate family-based obesity prevention interventions [12]. Identifying strategies to actively engage parents in such programs is a research priority for the field [10,13,14].

This paper presents case studies of published family-based interventions to provide insights and recommendations for researchers and practitioners. These considerations focus on four key areas: (i) study design and data collection; (ii) intervention development and implementation; (iii) recruitment; and (iv) engagement and retention. The paper was undertaken by members of the Parenting, Child Behaviour and Well-being Stream of the Australasian Child and Adolescent Obesity Research Network (ACAORN), who have considerable and varied expertise in conducting family-based obesity prevention interventions. Previous papers from the ACAORN group have provided recommendations and insights for researchers conducting school-based obesity prevention interventions [9] and obesity treatment interventions with overweight children [15]. The aims of this paper are:

- to highlight considerations regarding the design, implementation, and evaluation of obesity prevention interventions with families,
- to provide practical insights for researchers and practitioners to overcome some of the key challenges associated with testing family-based obesity prevention interventions, and
- to produce a summary list of recommendations for best practices in conducting obesity prevention interventions with families.

## 2. Overview of Studies

To support the study aims, we draw reference to three family-based obesity prevention randomized controlled trials (RCTs), which were successfully conducted by members of ACAORN. The NOURISH [16,17] and ‘Infant Feeding, Activity and Nutrition Trial’ (InFANT) [18,19] RCTs targeted mothers of infants/toddlers and the ‘Healthy Dads, Healthy Kids’ (HDHK) RCT [20,21,22] targeted fathers of primary school-aged children. Although these interventions targeted different combinations of family members, all can be considered ‘obesity prevention interventions’ as a primary goal of each was to minimise child weight gain or risk for obesity onset during the study. In addition to reporting child body mass index (BMI) z-score, all featured studies reported behavioural outcomes (e.g., physical activity, diet), familial/parenting outcomes (e.g., parenting beliefs and practices) and process evaluation outcomes (e.g., satisfaction, retention, attendance). These studies are briefly summarised below and additional details are provided in Table 1.

### 2.1. NOURISH

The NOURISH intervention provided anticipatory guidance to 698 first time mothers and included two modules of six 1–2 h interactive group sessions held fortnightly, delivered when the infant was aged 4 to 7 months and 13 to 16 months [16,17]. The first module focused on exposing infants to a wide range of tastes and textures to promote healthy food preferences and the second focused on responsive feeding (i.e., the recognition and appropriate response to hunger and satiety in order to promote self-regulation of energy intake relative to need). Both modules included content on positive parenting (warmth, encouragement of autonomy, and self-efficacy) [16] (Table 1).

Data from children and parents were collected at baseline and when children were aged 14 months and 2 years. Participant satisfaction was high and retention was 78% at 2 years (Table 1). Various strategies were used to ensure intervention quality and fidelity. These included the use of standardised training, procedural manual and presentation materials, fortnightly teleconference reviews between facilitators, and independent observation of 15% of sessions. At two years, significant group × time differences were observed in the majority of child feeding practices (e.g., responsive feeding), favouring intervention mothers, which were the primary outcome for the trial [17].

### 2.2. InFANT

The InFANT program is a 15-month program delivered quarterly (for children aged 3–18 months) in community settings to first time parent groups. By providing anticipatory support, the program aims to provide mothers with knowledge and skills to promote healthy eating, increase physical activity, and reduce sedentary behaviours in their children across the early years. The InFANT program was trialled in a cluster-randomised controlled trial [18,19] (see Table 1) and is currently being implemented by eight local government areas in Victoria (Australia). The translation of the program from RCT trial to community rollout is currently being evaluated.

Although participation in the InFANT Program did not affect child BMI z-score at 18 months of age, a range of important effects were demonstrated for secondary outcomes such as children’s television viewing time, children’s dietary quality, and maternal dietary patterns (see Table 1). Recruitment and retention rates were high, as were parent perceptions of program usefulness and relevance [19].

### 2.3. HDHK

HDHK is a 3-month program designed to help the fathers manage their weight and to role model healthy behaviours for their children. The program provides fathers with the knowledge and skills required to spend time with their children using healthy eating and physical activity as the engagement mechanisms. To date, HDHK has been tested in both an efficacy RCT [20] and an effectiveness RCT [21,22]. As seen in Table 1, program participation resulted in clinically significant weight loss for fathers, improved BMI z-score in children, and several other meaningful health improvements. Participant satisfaction was high and retention levels exceeded 80% in both trials.

## 3. Areas of Consideration for Obesity Prevention Research with Families

In the following sections, we will provide a series of insights, lessons learned, and best-practice principles to consider when designing, implementing, and evaluating family-based obesity-prevention interventions. Rather than focusing on general recommendations for conducting methodologically-sound behavioural research trials (e.g., ensuring adequate study power, adhering to randomization procedures), which can be located elsewhere [24], this paper has a distinct focus on the unique challenges of conducting obesity prevention trials with parents and families.

### 3.1. Study Design and Data Collection

Many obesity prevention interventions to date have used uncontrolled study designs [7], which limits inference about treatment efficacy. While the RCT is the most powerful research design for minimising bias and establishing causal effects, implementing RCTs in family-based research requires careful consideration of several important factors.

Randomising families to a no-intervention control group may affect the motivation of families to enrol or remain involved in the study. This can increase the risk of sampling bias and missing data, often disproportionally between groups. An alternative option for researchers to consider is the use of a wait-list control group, where control families are offered the program after data collection has completed. This option was used effectively in both HDHK trials, where control group retention rates were 92% and 87% at the end of the pilot [20] and community RCT [21], respectively. However, it should also be noted that a wait-list control design may not be practical in studies which target a very narrow age group, as was the case in InFANT and NOURISH. In these studies, researchers could offer the control group newsletters or other resources with information on non-obesity related issues relevant to families as an alternative.

While not utilised in our three featured studies, another option available to researchers is the attention placebo control group, where families receive advice on an unrelated behaviour (e.g., food safety, bullying), but are provided with the same amount of program sessions and interventionist support. Attention placebo groups can partially offset the inflated intervention effect that can be observed when using wait-list controls [25], but are difficult to implement appropriately in health behaviour research and require high-level consideration of methodical and ethical issues [26]. As such, researchers interested in using attention placebo controls are advised to access helpful information and guidelines elsewhere [25].

A further challenge of using an RCT design in family-based research is that researchers should ideally collect baseline data prior to randomisation. Although this is an important strategy to reduce bias, in a practical sense it requires families to make a firm commitment to the study without knowing which study arm they will be randomised into. This issue is not generally a concern in single-arm trials, but can be problematic in studies with multiple intervention arms that are delivered on different days of the week or in studies with wait-list controls. In our experience, we have found that these issues can be minimised by providing clear details to families about the importance of randomisation, ensuring they are available to participate in the program on any of the proposed nights, ensuring they understand that they are participating in a research trial rather than a community program, and explaining the valued contribution that control/comparison groups make to important research outcomes. In programs with wait-list control groups such as HDHK, we also emphasised to families at first contact (and prior to conducting baseline assessments) that they must be available to do the program immediately or at a pre-defined later date (e.g., term 1 or term 2) and this is listed as a key eligibility criterion.

In addition to selecting an appropriate research design, identifying which primary and secondary outcomes will be used to evaluate the intervention is another key consideration. In obesity prevention trials, weight-related outcomes are commonly primary (e.g., BMI, BMI *z*-score, percentage body fat, waist circumference). Given the sensitive nature of these measures, it is important that the measures are taken by trained assessors with appropriate clearances and experience working with children. This information should also be relayed to families to ensure they understand and are comfortable with the process.

Secondary measures should reflect the focus of the intervention and will usually include diet and activity outcomes as key influences on weight status. Given the growing body of evidence suggesting that parenting variables have important influences on child health behaviours [27], the inclusion of high quality measures of parenting practices and other family variables, such as the quality of the co-parenting relationship [28], are also recommended. As different family members have unique insights into child behaviour, researchers should consider capturing the perspectives of multiple family members. This practice also minimises the influence of common-methods bias, where systematic error is introduced by measuring variables through a single source or method. In line with the existing literature [29,30], HDHK data revealed significant differences between mother and father parenting practices for physical activity and nutrition and determined that both parents have unique influences on their children’s health [31]. Further, while mothers often complete self-reported measures of child behaviour, a recent study identified that child- and father-completed food frequency questionnaires provided more accurate estimates of child energy intake compared to those completed by mothers in some instances [32]. Although the sample size for the study was small (due to the expenses associated with use of doubly labelled water), it highlighted the need to ensure the insights of multiple family members are considered and/or included wherever possible when collecting self-reported data. 

Although research trials can investigate whether or not an obesity prevention program has worked, much less is currently known about which particular intervention components or mechanisms are most important to enable the intervention to achieve its intended effect, particularly in the context of parenting [12]. Measuring key behavioural variables (i.e., parenting practices) and psychological variables (i.e., parenting beliefs) with valid and reliable measures will allow for potential mediation and moderation analyses, which can help to answer this question. In the InFANT program, increased maternal knowledge of child feeding intervention messages and the reduced use of foods as rewards mediated the intervention effect on child diet quality [33]. This mediation analysis was also useful to show that most of the maternal beliefs and behaviours targeted in the intervention were associated with child diet quality, confirming that the targets were appropriate [33]. Similarly, mediation analyses of the HDHK program have revealed that increases in physical activity (i.e., steps/day) and co-physical activity (i.e., shared physical activity between fathers and children) significantly mediated the program’s effect on fathers’ weight loss [34] and children’s physical activity [35], respectively. This information was then used to refine the program, which now includes more interactive sessions for fathers and their children, and a greater focus on completing fun, home-based physical activity tasks and games.

Process evaluation is also important in any intervention to assess acceptability and relevance to target population and inform program modification to maximise generalisability and translation [36]. Major components of process evaluation include program reach to ensure the most appropriate end user is being targeted, participant satisfaction, acceptability or understanding of program materials, and implementation data in terms of attendance, engagement, and fidelity (i.e., whether the components of the research program are being implemented as intended). In family-based research, it is imperative to consider multiple perspectives from multiple stakeholders, as mothers, fathers, children, and facilitators will all have unique and important insights and perspectives that can be helpful when refining and improving the program.

### 3.2. Intervention Development and Implementation

As the role of parenting is dynamic over time and needs to adapt as children grow older, family-based obesity-prevention programs should ideally provide parents with skills that are transferrable across a range of child developmental stages. This is particularly important given recent reviews that have indicated that parenting style and parenting practices have important influences on children’s health behaviours and obesity risk [27,37]. All of our featured studies have explicitly addressed these issues by providing parents with transferrable skills in areas including, for example, parent feeding, limit setting, creating family rules, and following an authoritative parenting style [17,19,20,21].

In line with most behavioural interventions, family-based obesity prevention programs are often complex and contain a large and varied number of different cognitive and behavioural strategies. Of concern, few papers include sufficient information on these intervention components to allow for replication studies or systematic investigation of the most effective techniques across programs [38]. In their formal taxonomy of behaviour change techniques (BCTs) (e.g., self-monitoring of behaviour), Abraham and Michie have provided an efficient way for researchers to provide this information in their reports [39]. Notably, a recent systematic review of parent interventions to improve children’s diet and physical activity behaviours collated the reported evidence on BCTs and identified a series of techniques linked to effectiveness [12]. These BCTs, which included specific goal setting, barrier identification, graded tasks, self-monitoring, and restructuring the home environment, should be given important consideration by researchers deciding on program components in future interventions targeting families.

Facilitator training is another critical element of intervention development and delivery. Indeed, the quality of the program facilitator has an important moderating influence on the effectiveness of any program [40]. In the InFANT study, facilitator training involved meetings every 3 months with all facilitators and the lead investigators across the trial period (15 months). This allowed the incorporation of facilitators’ experience, opportunities for increasing program ownership, and re-enforcement of processes, procedures required to maintain program fidelity and study requirements. Alternatively, in the NOURISH study, facilitators attended an intensive, single-day training workshop. This workshop covered the theoretical underpinnings of the intervention, an overview of the content of each session, how to manage group dynamics and respond to questions (particularly recognising limits of expertise), and an opportunity to role play the delivery of sessions. Facilitators were provided with session checklists to help them keep to schedule and ensure rigour. They were also encouraged to debrief with study staff and each other about aspects of program delivery that worked well or were difficult.

In addition to the content knowledge and qualifications of the program facilitators, successful program implementation relies on the confidence of the facilitator, their communication skills and the training and experience they have in delivering ‘best practice’ teaching strategies [40]. In our studies we have integrated many novel pedagogical delivery methods, such as those outlined in the Productive Pedagogy and Quality Teaching frameworks [41] to maximise familial engagement during program implementation. For example, to address the important pedagogical technique of narrative or ‘story telling’, HDHK includes ‘built-in’ opportunities for facilitators to share their own stories (or those of previous participants) about occasions where implementing the program recommendations, while often challenging, has led to improvements in their health or family life. Similarly, in InFANT, parents are encouraged to share new achievements with the group (such as trying a new vegetable and discussing how they cooked it). In our experience, we have found that parental engagement is enhanced when the intervention sessions are facilitated by confident and competent professionals, who: (i) are familiar with teaching and behaviour change techniques; (ii) are skilled in managing group dynamics; (iii) have significant experience and expertise in the focus areas of the intervention (i.e., physical activity, diet modification, sedentary behaviour, etc.); and (iv) are comfortable with managing groups of children, if they are also participating in the sessions.

### 3.3. Recruitment

Recruiting families into prevention interventions can be challenging [42]. It is important to note that the recruitment approach used will depend on a number of factors related to the target population and the intervention design, including: the age of children in the families; socio-economic status; cultural factors (e.g., ethnicity, language); availability of potential target groups within the community (e.g., early childhood groups, schools); type of intervention (e.g., individual or group sessions); setting of intervention delivery (e.g., clinic, community-based, online), and any other factors specific to the target group and environment within which recruitment and the project will be undertaken. Within these trial-specific contingencies, there are a number of general concepts for consideration.

A key reason that parents do not enrol in family-based obesity prevention studies is often a perceived lack of program relevance. Indeed, research has demonstrated that parents are often unaware that their child is at risk for becoming obese [43] and many incorrectly believe their children are meeting recommendations for physical activity, healthy eating, and screen time [44,45]. As such, recruiting based on a premise of general improvements in child weight status, healthy eating habits, or physical activity levels may not be a particularly effective strategy [46,47]. We have found that specifically focusing on common challenges rather than broad behaviours can somewhat offset this problem (e.g., getting kids away from screens, preventing family dinners from becoming ‘battlegrounds’). Alternatively, recruitment material could focus on improvements in psychosocial outcomes (e.g., quality of life, social-emotional well-being, self-esteem, body image, spending time together, enjoyment) rather than physical and behavioural outcomes. Promotion of the psychosocial benefits of physical activity may also facilitate the recruitment of families usually stigmatized by obesity [48]. In our research, the HDHK program was targeted to fathers as an opportunityto spend quality time with their children and improve their social-emotional wellbeing through participation in fun rough and tumble and sports-based physical activities. Alternatively, the InFANT and NOURISH programs focused on ‘getting healthy eating and active play right from the start’ and optimising ‘your child’s future health, today’, which tapped into the desire of first-time parents to learn more about how to feed their children and establish healthy behaviours from a young age.

Another useful recruitment strategy to consider is targeting common meeting places for the study sample. The NOURISH trial recruited first time mothers via maternity wards. Similarly, InFANT recruited mothers through existing mother and babies groups established through Maternal and Child Health services. It may also be useful to target extracurricular activities (e.g., swim, dance schools, play groups, music schools). Once embedded in these settings, it is recommended to engage with existing staff to promote the program to their clients. For example, community liaison workers and indigenous support workers can be employed in schools, local councils, and youth clubs. Midwives can be employed to recruit mothers and this was an effective strategy utilised in NOURISH. In addition, specialist centres, such as those targeting low-income families or families of a specific ethnicity, may be appropriate if these suit the needs of the research project.

Education settings are a particularly important setting for recruitment for all ages. A starting point is to contact school principals/administrators and inform them of the study and its benefits for the school community. Examples of school-based recruitment strategies include school newsletters or fliers, parent-information nights, or approaching parents at pick-up and drop-off times. Of the numerous strategies used to recruit parents into the HDHK study [22], those associated with schools were most successful. The key strategies were face-to-face interactions at pick-up times, advertisements in school newsletters, and presentations to students at school assemblies from school champions or research staff [20,21]. With the appropriate ethics approval, recruiting staff members as site-specific recruitment ‘champions’ can be invaluable.

Parents also need to feel as though their involvement in an intervention will cause minimal disruption to their family schedule. Thus, it is important to recognise and anticipate challenges to participation by families and account for these during the recruitment process. To address this, the InFANT trial intervention was advertised as a program that would be delivered to first time mothers’ groups at times when they would normally be meeting. Our programs have also attempted to reduce possible disruption by offering free childcare during session times, providing flexibility in the selection of times and dates for meetings, and provided reimbursement for travel to regional mothers [49].

Given the challenges inherent in recruiting families, strategies to improve recruitment should target potential participants via multiple avenues on multiple occasions. Notably, parents were more likely to enquire about enrolling in the NOURISH program after they had seen recruitment material in multiple sites such as a letter in the school newsletter, a local newspaper advertisement, and a television news report.

### 3.4. Engagement and Retention

Engaging and retaining families in obesity prevention interventions is another key challenge that is central to intervention success [10,12]. Understanding who and how best to target family members is fundamentally important.

Utilising a Community Based Participatory Research (CBPR) approach has been suggested as an ideal method of optimising parental/familial engagement in obesity prevention programs [50,51]. This approach promotes the engagement of parents throughout the entire research process (i.e., from the formative phase to the evaluation phase), rather than just during the implementation phase. The CBPR approach aims to foster parental empowerment and encourage co-learning between all stakeholders [52]. Furthermore, it promotes open communication, breaks down hierarchical relationships, and builds trust between researchers and parents. Sustained active participation of parents results in a more salient, culturally responsive, and sustained intervention [52]. Our studies have utilised the CBPR approach by engaging families, where possible, in all phases of the research process. For example, as a prelude to the development of the InFANT program, parents, and ‘Maternal and Child Health Nurses’ were interviewed regarding their attitudes, beliefs, and perceptions regarding healthy eating and active play for children, which provided key insights that were used during the development of the intervention. Similarly, aspects of the NOURISH study (specifically regarding program content, frequency and method of delivery, and the collection of evaluation data) were informed by the experiences of the parents who participated in the pilot study, titled “Nourish to Flourish”. Their recommendations were provided via focus group discussions held after the completion of the pilot. Health professionals and key organisations also provided guidance and advice through membership of a Project Steering Committee. Key advice provided from this Committee members included program content and recruitment strategies to ensure their support during the recruitment and implementation phases of the study.

Although our featured studies predominantly targeted one parent, making efforts to engage other family members is also important. In our studies, we have used a number of strategies to engage these ‘other’ family members. For example, in NOURISH, resources were developed for extended family members (e.g., grandparents, fathers) which communicated program messages. In the InFANT program, all resources were presented on a DVD (now freely accessible online at www.infantprogram.org), which the participating parents were encouraged to show to other carers, including the non-participating parent and grandparents. Although HDHK specifically targeted fathers, mothers were invited to two of the group sessions and were provided with a mothers’ handbook, which gave an overview of each session and what their partners and children would be learning about each week.

The capacity of families to engage with the intervention may be further enhanced by offering alternate methods of delivery and reinforcing that missing some sessions does not mean families have to withdraw from the program. Based on our experience, delivering programs face-to-face is a useful way to maintain engagement with families through the study. However, advances in e-health delivery modes (e.g., web- or mobile-based programs) have allowed for the development of engaging, distance-based programs that minimise participant burden and increase program scalability. It is also important to offer flexible delivery modes for those who are unable to attend the face-to-face meetings or can only attend some of the sessions (e.g., parents who work full-time or parents who have unusual work schedules such as shift workers). For example, families who missed sessions in the InFANT study were given the resources to cover the program content and followed up with a phone call to check understanding. This complementary mode of delivery ensured that all parents could access the program content irrespective of their weekly schedule.

Often there is a lag time between study recruitment and program implementation due to the need for the recruitment of adequate group numbers. It is important that parents are kept engaged during this period to ensure that their “readiness to change” does not wane [53]. To keep InFANT participants engaged in the 3-month period between the first and second session, a newsletter was mailed that repeated key program messages. Targeting existing social groups in the study also encouraged participants to support each other and discuss program recommendations between sessions.

Maintaining parental engagement after the intervention concludes is also imperative if the study design includes additional follow-up assessments. During this time, the lack of regular contact with facilitators and/or researchers may contribute to disengagement from the study. Collecting the mobile phone numbers and email addresses of all caregivers (and possibly an additional family member or close friend) at the beginning of the study is a useful strategy to reduce the chances of losing contact with participants during this time. In the NOURISH study, mothers were given postcards and reply paid envelopes to remind them to inform the study team if they moved or changed contact details [49]. To maximise retention, mothers who moved during the study period were provided with instructions for taking their child’s physical measurements and reply paid envelopes for returning questionnaires. Other strategies we have used to maintain contact with families between assessments include newsletters, emails, social media accounts, and Christmas and birthday cards. We have also found that the use of automated SMS messages to remind parents about upcoming sessions and assessments is very helpful to maximise study retention.

Minimising participant burden and making it as easy as possible for families to fulfil their research commitments are particularly important considerations to maximise retention. Strategies to reduce burden in our studies include: having all of the measures conducted at a convenient time and place, having blood collectors onsite, and offering alternate options (e.g., multiple assessments per week, multiple appointment times for each day). In HDHK, fathers also had the option to complete questionnaires online at home prior to the assessment to minimise time spent filling in questionnaires at the session. After exhausting all options to have families attend one of the scheduled assessment sessions, our programs also provided families with the option of a home visit to complete the assessment.

## 4. Conclusions

Obesity is associated with a wide range of negative health consequences [54] and, once established, is very difficult to reverse [55]. As such, the international increase in the prevalence of childhood overweight and obesity [1] is a major public health concern that requires an urgent solution. While behavioural obesity prevention programs are an important part of this solution, the effect of existing programs has been modest and most have failed to actively engage parents [7], who have a fundamental role in modelling and establishing healthy lifestyle behaviours in their children. As such, researchers have called for more studies to actively involve parents in future programs [10,13,14,56,57].

This article has provided a series of practical insights for researchers who plan to conduct childhood obesity prevention intervention research with parents and families (for a summary of recommendations, see Table 2). Although these insights were provided by researchers across five institutions, all studies were conducted in Australia. As such, we encourage other researchers to contribute to the field by sharing additional insights gained from research conducted in other settings and countries. When combined with qualitative and quantitative evidence, these experiential insights may help researchers to achieve a greater and more meaningful representation of parents in childhood obesity prevention research internationally.

## Figures and Tables

**Table 1:** Summary of family-based childhood obesity prevention randomized controlled trials.

Study, Primary Aim	Sites and Sample	Study Arms	Assessments, Retention	Results
**NOURISH [16,17]** **RCT** *Primary Aim* To evaluate an intervention promoting protective feeding practices to prevent childhood obesity.	*Site(s)* Community child health clinics *Sample* 698 first time mothers (mean (SD) age = 30.1 (5.3) years) with healthy term infants (51% female; mean (SD) age = 4.1 (1.0) months).	*Intervention* Participants received two modules starting when the children were aged 4–7 months and 13–16 months, respectively. Each module included six group sessions over 12 weeks. Program provided anticipatory guidance to parents (i.e., pre-emptive information and constructive advice about established problems with child eating behaviour and weight status). *Comparator* Usual care *Theory* Attachment theory, anticipatory guidance, SCT	*Assessments:* Baseline (infant 4-7 month), 9 month from baseline (~6 month post module 1, infant 14 month), 20 month post baseline (~9 month post module 2, infant 24 month). *Retention* 9 month: 86% 20 month: 78%	*Primary outcome* Significant treatment effects favouring the intervention group detected at 20 months for several parent feeding practices including: pressure to eat (*p* < 0.001), instrumental feeding (*p* < 0.001), encouragement (*p* = 0.01), and emotional feeding (*p* = 0.04) [17]. *Secondary outcomes* No statistically significant differences were detected for child BMI z-score (MD (SE) = −0.14 (0.09), *p* = 0.10) or prevalence of overweight/obesity (control 17.9% vs. intervention 13.8%, *p* = 0.23) [17]. *Example process outcomes* Attendance at ≥2 sessions for module 1 was *n* = 229 (65%) and module 2 was *n* = 130 (45% of those retained at module commencement) [17].
**InFANT [18,19]** **Cluster RCT** *Primary Aim* To establish the efficacy of an intervention targeting first-time parents to prevent obesity in children by improving dietary, physical activity, and screen time behaviours.	*Site(s)* 52 community-based first-time parents groups. *Sample* *n* = 542 mothers (mean (SD) age = 32.3 (4.3) years), with healthy term infants (47% female; mean (SD) age: 3.9 (1.6) months.	*Intervention* Six group sessions over 15 months, supported by take-home DVD and written resources. Program focused on ways to improve child nutrition, physical activity and sedentary behaviours, parental modelling, and appropriate feeding practices. *Comparator* Usual care plus six newsletters on unrelated general health topics. *Theory*: Anticipatory Guidance, SCT	*Assessments* Baseline (child 4 months), mid-intervention (child 9 months) and post-test (child 20 months). *Retention* Mid-intervention: 94% Post-test: 89%	*Primary outcome* No treatment effect on child BMI z score at 18 months of age. *Secondary outcomes* Significant treatment effects favouring intervention for child noncore drink consumption (MD = −4.5; 95% CI: −7.9 to −1.0; *p* = 0.01) [19], child’s sweet snack consumption (MD = −3.7; 95% CI: −6.4 to −1.0; *p* = 0.008) [19], child TV viewing time (MD = −16.0: 95% CI: −26.0 to −6.0; *p* = 0.002) [19], maternal intake of high-energy snacks/processed foods (MD: −0.2 (−0.4; −0.0)) and high-fat foods (MD: −0.3 (−0.5; −0.0)) [23]. Significant treatment effect also identified for maternal knowledge and feeding practices (potential mediators of intervention effectiveness). Intervention arm reported: higher maternal knowledge of child feeding intervention messages (α: 1.0; 95% CI: 0.6 to 1.4), higher intentional modelling of healthy eating (α: 0.5; 95% CI: 0.0 to 1.0), lower use of foods as rewards (α: −0.8; 95% CI: −1.5 to −0.1) and lower use of pressure in feeding (α: −0.5; 95% CI: −1.0 to −0.0). *Example process outcomes* 89% of intervention participants completed the trial and of these 68% attended the majority of intervention sessions (≥4 of 6 sessions), whereas just 9% attended <2 sessions. Mothers consistently reported high levels of program usefulness and relevance [19].
**HEALTHY DADS,** **HEALTHY KIDS** **(a) Pilot RCT [20]** **(b) Community RCT [21,22]** *Primary Aim* (a) Test the efficacy of the HDHK program to help overweight fathers lose weight and positively influence the health behaviours of their children. (b) Test the effectiveness of the HDHK program to replicate initial program effects when delivered by trained facilitators in regional, disadvantaged communities.	*Site(s)* (a) University (b) Primary schools *Sample* (a) 53 overweight/obese men (mean (SD) age = 40.6 (7.1) years) and their primary school aged children (*n* = 71, 46% female; mean (SD) age = 8.2 (2.0) years). (b) 93 overweight/obese fathers (mean (SD) age = 40.3 (5.3) years) and their primary school aged children (*n* = 132; 45% female; mean (SD) age = 8.1 (2.1) years).	*Intervention* (a) Fathers attended eight 90-min face-to-face group sessions (three with their children) over 3 months and received program resources (booklets, pedometers). The program was delivered by study chief investigators. (b) Fathers attended seven 90-min face-to-face group sessions (three with their children) over 3 months and received program resources (booklets, pedometers). The intervention was delivered by trained local facilitators. *Comparator* (a and b) Wait list control group *Theory:* (a and b) SCT and Family Systems Theory	*Assessments* (a) Baseline, post-test (3 months) and follow-up (6 months). (b) Baseline and post-test (3 months). *Retention* (a) 3 months: 83% 6 months: 83% (b) 3 months: 84%	*Primary outcome* (a) Significant group × time difference at 6 months (d = 0.5) with HDHK fathers losing more weight (−7.6 kg) than control fathers (0.0 kg). (b) Significant group × time difference at 3 months (d = 0.2) with HDHK fathers losing more weight (−3.3 kg) than control fathers (0.1 kg). *Secondary outcomes* (a) Significant treatment effects were also found for fathers’ waist (d = 0.6), BMI (d = 0.5), systolic blood pressure (d = 0.9), resting heart rate (d = 0.7), and physical activity (d = 0.9), but not for dietary intake. In children, significant treatment effects were found for physical activity (d = 0.7), resting heart rate (d = 0.5), and dietary intake (d = 0.8). (b) Significant effects were also found for fathers’ waist (d = 0.4), BMI (d = 0.3), resting pulse (d = 0.6), energy intake (d = 0.5), and physical activity (d = 0.5) and for child activity (d = 0.5) and BMI z-score (d = 0.1). *Example process outcomes* (a) Mean attendance: 81%. Program satisfaction (mean: 4.8/5, SD: 0.4); facilitator quality (mean: 4.9/5, SD: 0.2). (b) Mean attendance: 71%. Program satisfaction: (mean: 4.8/5, SD: 0.4); facilitator quality (mean: 4.3/5, SD: 0.5).

MD, mean difference; CI, confidence interval; d, Cohen’s d; InFANT, Infant Feeding, Activity and Nutrition Trial; HDHK, Healthy Dads, Healthy Kids; RCT, randomised controlled trial; SCT, social cognitive theory; BMI, body mass index; TV, television.

**Table 2:** Recommendations and considerations for conducting childhood obesity prevention research studies with families.

Study design and data collection	Utilise a randomised controlled design to establish program efficacy wherever possible. Consider using wait-list or attention placebo control groups to reduce ethical concerns and minimise control group dissatisfaction and dropout. Ensure families understand they are registering for a university trial rather than a community program (explain the importance of this research component). In addition to measuring child BMI z-score, carefully select secondary measures to establish the full range of program effects for families. Ideally, these measures should cover the following domains: behavioural (physical activity, diet), psychological (e.g., quality of life, body image), parenting practices and beliefs (e.g., co-physical activity, use of food as a reward) and process evaluation (e.g., satisfaction, attendance, fidelity). Give consideration to which family members will complete child proxy measures. Collect multiple perspectives (e.g., mothers, fathers) where possible.
Intervention development and implementation	Test intervention components, structure and delivery mode in a pilot trial. Integrate insights from parenting literature and operationalise constructs from parenting theories (e.g., family systems theory) for improved health behaviour outcomes. Select behaviour change techniques that have been linked to increased program effectiveness in previous family-based obesity-prevention programs (e.g., barrier implementation, restructuring home environment). Choose a mode of delivery that engages both parents and children in age appropriate activities. Consider provision of child-care for parent-only sessions to reduce burden. In addition to face-to-face delivery, consider integrating alternative delivery modes (e.g., web- or mobile phone-based) to increase scalability and minimise participant burden. If the program targets one parent only, provide take-home resources to ensure other family members have access to the material. Recruit confident and competent program facilitators who have expertise and experience implementing best practice teaching strategies relating to family based interventions.
Recruitment	Target valued parental outcomes in recruitment materials. Focus on psycho-social and emotional benefits rather than physical outcomes. Minimise program components that are likely to disrupt established family routines. Utilise existing social groups or places where parents are gathering for other reasons (e.g., schools) Target parents multiple times using multiple sources.
Engagement and retention	Involve families in all aspects of program design and implementation. Employ strategies to ensure all family members are ‘on the same page’ and reduce the potential for tension when new strategies and behaviours are trialled at home. Provide families with flexibility in program delivery. Maintain contact with families between recruitment and program delivery and during post-intervention follow-up periods. Collect mobile phone numbers and email addresses of several family members to reduce possibility of losing contact. Use SMS reminders in the lead up to assessments (and on the day) to maximise attendance.
